# Purulent Pleurisy*Address as retiring President, delivered at April meeting Taylor County Medical Society.

**Published:** 1892-06

**Authors:** W. W. Wallace

**Affiliations:** Abilene, Tex.


For Daniel's Texas Medical Journal.

### PURULENT PLEURISY.*

BY W. W. WALLACE, M. D., ABILENE, TEX.

GENTLEMEN :—In retiring from the position with which you have honored me—that of presiding officer of Taylor County Medical Society for the past year—I shall not trespass upon your time by an elaborate essay on matters non-medical, but shall simply endeavor to call to your attention a subject of medical and surgical interest, and briefly to refer to a case illustrative of the subject, which recently occurred in my practice. The subject selected by me is

### PURULENT PLEURISY.

The term purulent pleurisy should be preferred, as it more clearly and fully expresses the pathological condition—inflammation of the pleura with effusion or formation of pus. Other terms are sometimes used, such as empyema and pyothorax. I shall confine my remarks to the disease as it occurs from general causes or is secondary to other diseases. Some believe the effusion at first to be serous, and afterwards purulent from degeneration or intensity of inflammation, though in some cases it is undoubtedly purulent from the first, not simply from a deposit of pus, but following, and a result of a more or less intense inflammation of the pleura.

Some authors believe the fluid in all pleurisies to contain pus globules, but Wilson Fox says, "there is but little natural tendency in serous effusions to undergo purulent transformations," and thinks that in the majority of cases the fluid is purulent at an early period of the disease.

Purulent pleurisy may follow any of the exanthemata, such as variola, scarlatina and rubeola, and it is said that pleurisy in the puerperal condition is nearly always purulent. It is often purulent after severe injuries other than those of the pleura, and following exhaustion or lowering of the vital forces from overwork, excesses and protracted diseases. It is often difficult, if not impossible, to explain how a serous effusion following acute pleuritis may later on become purulent, but a chronic pleurisy is

*Address as retiring President, delivered at April meeting Taylor County Medical Society.

generally purulent, yet it may be that a correct diagnosis was not made at an earlier period of the disease. Men are more liable to this disease than women, and children more so than adults.

I was called during the night of December 9, 1891, to see Julian McLemore, age 16. He had for perhaps a week suffered from an ordinary cold, with cough, etc., and a few hours previous to sending for me had been seized with a chill and violent pain in the left side. There was high temperature and intense pain on the following and succeeding days, except when controlled or modified by opiates and antipyretics. By physical signs, and the appearance of profuse rust-colored expectoration, I was soon convinced that I had on hand a typical case of pneumonia. But it is not my purpose to dwell upon this part of the clinical history of the case, and will simply state that the pneumonia symptoms gradually disappeared, and the temperature fell below 100° F. and so remained for three or four days, but pain in the side still continued and was a prominent symptom throughout the entire course of the disease. By increase of pain and rise of temperature I was of the opinion for a short time that the pneumonic process had extended to another lobe of the lung, but auscultation and percussion failed to confirm me in that belief. Careful investigation and search was made for complications, The temperature often went up to 104°, 104½° and on one occasion to 105°. About the middle of the third week from the inception of the disease the physical signs indicated very plainly a fluid collection in the plural cavity, and the aspirator established the diagnosis of purulent pleurisy. With Allen's surgical pump a halt pint of that pus was withdrawn when the needle became clogged. This instrument and other expirators were repeatedly used but with indifferent success, owing to the density of the pus and the presence of numerous coagula. Finally I determined to make an opening sufficiently large to freely evacuate the purulent contents, maintain drainage, and to enable me to thoroughly wash out the pleural cavity. The inhalation of chloroform or ether being out of the question here, I introduced a 5 per cent. solution of cocaine hypodermically around and at the point selected for the incision.

The skin was bathed with a bichloride of mercury solution 1 to 1,000, all instruments and sponges thoroughly disinfected, and every precaution used to insure perfect asepsis and antisepsis. The side was so œdematous as to entirely obliterate the intercostal spaces and to conceal the ribs, which made it very difficult to accurately select a given point for operation. Therefore I made an incision downward and forward, beginning at the median axillary line, to the extent of an inch, and carefully divided, layer by layer, using the index finger of the left hand as a guide, until the upper border of the seventh rib was reached. The intercostal space was too narrow here to give entrance to a drainage tube of sufficient calibre, and I put the point of a large-sized trocar against the upper margin of the rib, and cut a shallow groove through it, which was afterwards distinctly felt and recognized by the finger. A metal canula was introduced, and left in for twenty-four hours, and afterwards, at different times, a section of hard rubber catheter. Nelaton's soft catheter or rubber tubing was used as a drainage tube, and through which the parts involved in the morbid process could be irrigated with antiseptic solutions. Once in every twenty-four hours the dressings were changed, the side bathed with bichloride solution, and bichloride gauze and bichloride cotton packed over and around the wound for a distance of six inches or more; and over these dressings was placed a piece of oil cloth previously dipped in a bichloride solution, and all kept in place by a clean bandage passed around the body. The discharge of pus was very profuse, and it frequently became necessary to remove the tube because of its becoming clogged, and to cleanse and reintroduce it, or substitute a new one. The cavity of the pleura was washed out many times with a solution of resoıcin, 10 grains to half a pint of warm water, by means of a soft rubber catheter used as a siphon or attached to Allen's surgical pump or a smaller aspirator, which I found worked more thoroughly and satisfactorily. The pleural cavity was irrigated until the water returned clear. Under this treatment, the temperature gradually fell to normal, profuse perspiration, pains and tenderness disappeared, and the appetite improved. As a precautionary measure, a short, soft rubber tube was left in for a week after pus had ceased to flow, and the resorcin solution used daily. After removing the tube a tent of bichloride gauze was used for three or four days more. Until complete closure of the wound, it was kept covered with antiseptic dressings.

At this time the young man is apparently restored to perfect health, but with some flattening of the side, yet there is no marked deformity. There is no disease which more surely tends to a fatal termination, if left to the resources of nature, than purulent pleurisy. Here the physician must act with promptness and energy, and I am decidedly of the opinion that the "open method"—evacuation of the pus by a free opening in the chest wall, with the use of antiseptics, will give better results than aspiration. By the "open method" with thorough asepsis and antisepsis, the danger of septicemia is reduced to a minimum. I have met with only four well-marked cases of this disease—one notably in which the pus was distinctly localized or encysted.

I was kindly and ably assisted by Dr. Blakemore.

